# The development and validation of the health literacy questionnaire for kindergarten teachers

**DOI:** 10.3389/fpubh.2025.1414277

**Published:** 2025-03-10

**Authors:** Han Ying, Yang DeHua, Ou Yang Yi, Wu Song-Wei

**Affiliations:** ^1^College of Teacher Education, Aba Teacher College, Aba, Sichuan, China; ^2^College of Education, West China Normal University, Nanchong, Sichuan, China; ^3^Chengdu University of Arts and Sciences, Chengdu, China

**Keywords:** kindergarten teachers, health literacy, reliability, validity, health education

## Abstract

**Background:**

Health literacy profoundly influences individuals’ health development. As pivotal figures in shaping young children’s well-being and delivering health education in kindergartens, kindergarten teachers are essential. Yet, assessing their health literacy remains challenging due to a scarcity of evaluation tools.

**Methods:**

Based on existing research, the initial questionnaire was developed through interviews, summaries, and reviews. A total of *N* = 120 (*M*_age_ = 27.19, *SD* = 6.75, 94.2% female) kindergarten teachers participated in item analysis and exploratory factor analysis (EFA). *N* = 642 (*M*_age_ = 28.12, *SD* = 5.77, 89.7% female) kindergarten teachers were involved in confirmatory factor analysis (CFA) and reliability analysis.

**Results:**

The questionnaire on the health literacy of kindergarten teachers consists of 30 items in four dimensions: *health concept*, *health behavior*, *health ability*, and *health knowledge*. In the EFA, the cumulative variance contribution rate reached 61.220%. The CFA indicators satisfied the fit criteria, indicating a well-fitted model (χ^2^/*df* = 1.945, CFI = 0.956, TLI = 0.952, SRMR = 0.034, RMSEA = 0.038). The reliability analysis indicated that Cronbach’s *α*, McDonald’s *ω*, and split-half reliability all exceeded 0.8.

**Conclusion:**

The Health Literacy Questionnaire for kindergarten teachers, with its strong reliability and validity, serves as a valuable assessment tool for this group’s health literacy.

## 1 Introduction

Health literacy was first proposed in 1974 by Simonds, an American scholar. Simonds posited that health literacy encompasses the ability of individuals to obtain, comprehend, and process fundamental health information, utilize health knowledge and technology, and make informed decisions conducive to their own well-being. Consequently, he contended that health literacy represents the amalgamation of an individual’s health knowledge and technology (1). Studies have indicated that individuals with varying characteristics experience distinct health issues, and as a result, their levels of health literacy may also differ (2–4). The unique role of teachers necessitates their acquisition, comprehension, and application of fundamental health information in their daily lives. Furthermore, it requires them to integrate this basic health knowledge into educational and instructional activities in order to enhance the health literacy level of their students. As a result, teachers’ health literacy should encompass both the general public’s health literacy as well as the specific professional health literacy required for their role as educators (5, 6). Therefore, Fred, an American scholar, proposed that teachers’ health literacy should encompass not only their ability to acquire, understand and internalize basic health information and services but also their capacity to apply this knowledge and these services to education and teaching in order to enhance the health status of those they educate (7). Chinese scholar Li Lin argues that teachers’ health literacy encompasses not only the fundamental knowledge required to maintain and enhance their own well-being, but also the capacity to positively influence students’ understanding of health, their knowledge about health-related matters, and their behaviors related to health. This includes three specific components: health concepts, health knowledge, and health behavior (6).

In conclusion, this study defines kindergarten teachers’ health literacy as a comprehensive quality in which teachers acquire, understand, and internalize health information in their daily lives and studies. They are able to organize and utilize this health information to maintain or promote their own and children’s physical and mental well-being. Specifically, it consists of four aspects: health concept, health knowledge, health behavior, and health ability. Among these aspects, the internalization of the health concept serves as the motivation; the acquisition of health knowledge acts as the fulcrum; mastery of health ability provides support; and application of health behavior serves as the purpose. It is worth noting that the health literacy formed by kindergarten teachers not only has a profound impact on their own career development, but also plays an essential role in enhancing overall well-being and quality of life (4, 8). Some studies have indicated that teachers with a high level of health literacy can more effectively promote the healthy growth of young children and demonstrate a more professional and confident demeanor in educational practice (9, 10). In addition, health literacy can help teachers better cope with work-related stress and challenges, improving their work efficiency and job satisfaction (10).

The development of effective health literacy measurement tools is not only crucial for supporting research in related fields but also holds significant practical importance for evaluating health literacy. Domestic and international scholars have developed various measurement tools for health literacy. The main ones are based on health-related fields, such as the Public Health Literacy Knowledge Scale (BHLKC) (11) and the Mental Health Literacy Scale (MHLS) (12), which can be utilized as an integral part of overall health literacy assessment. Additionally, there are health competency-based measuring tools, such as Zambia’s Health Literacy Scale (ZHLC) (13). There are also measures based on a combination of two models, such as the European Health Literacy Questionnaire (LS-EU-Q) (14), which was developed from a public health perspective. It is based on the four basic competencies (access, understand, evaluate, and use health-related information) and the three health-related areas (healthcare, disease prevention, health promotion). The questionnaire has demonstrated good reliability. In 2008, China’s Ministry of Health organized the first Survey of Chinese Residents’ Health Literacy and compiled the questionnaire of Chinese Citizens’ Health Literacy. This questionnaire is widely used across the country to measure the most fundamental health-related knowledge, ideas and skills that the general public must understand or master in their daily lives (15, 16). Later, Liu Hongyan developed the “Chongqing Adult Health Literacy Assessment Questionnaire,” which consists of 19 items categorized into four dimensions: health knowledge, health concept, healthy lifestyle, and health skills.

In conclusion, scholars have extensively discussed the measurement of health literacy, which serves as a valuable reference for scientifically assessing individual health literacy. However, several issues need to be addressed. Firstly, while foreign health literacy assessment tools are relatively well-developed and tailored to the characteristics of different populations from the perspectives of public health and clinical medicine, there is a lack of measurement tools applicable to Chinese cultural background. Secondly, while most of the health literacy tools developed by domestic researchers are applicable to general citizens, there is a lack of attention paid to teachers as a special group. Additionally, the existing health literacy measurement tools for teachers primarily focus on primary and secondary school teachers (17–19) with a noticeable gap in assessment tools for kindergarten teachers. The “Kindergarten Education Guidelines (Trial)” clearly emphasize the importance of protecting children’s lives and promoting their health as top priorities in kindergartens (20). As influencers of children’s physical and mental development and as implementers of children’s health education, the level of health literacy among kindergarten teachers has a significant impact on the healthy development of children, both physically and mentally. Furthermore, it also plays a crucial role in improving the overall quality of health education in kindergartens (21, 22). Therefore, the development of assessment tools for kindergarten teachers’ health literacy within the cultural context of our country has emerged as a pivotal focus in future health literacy research. Consequently, this study has compiled a questionnaire on kindergarten teachers’ health literacy under the current social and cultural backdrop of our country, aiming to provide scholars with a reliable and effective tool for measuring kindergarten teachers’ health literacy.

## 2 Methods

### 2.1 Participants and procedures

Sample 1: In November 2021, a paper-based questionnaire survey was conducted using offline random sampling method for kindergarten teachers from Chengdu and Mianyang. A total of 130 questionnaires were distributed in this survey. After excluding 10 invalid ones, the remaining valid sample size is *N* = 120 (*M*_age_ = 27.19 *SD* = 6.75, 94.2% female), with an efficacy rate of 92.31%. Among the participants, the demographic distribution was as follows: 110 identified as Han Chinese, with the remaining 10 belonging to ethnic minorities. The sample included 60 teachers from public kindergartens and an equal number from private institutions. In terms of teaching experience, 44 had less than 1 year, 54 had between 2 and 5 years, 6 and 10 years of experience was reported by 13 respondents, and 9 had over 11 years in the field. Regarding academic background, 95 specialized in early childhood education, while the remaining 25 held degrees in various other fields related to kindergarten education (see Table 1).

**Table 1:** Basic characteristics of kindergarten teachers as participants.

Variable		Sample 1 *N* = 120 (%)	Sample 2 *N* = 642 (%)
Gender	Male	7 (5.8%)	66 (10.3%)
	Female	113 (94.2%)	576 (89.7%)
Ethnicity	Han Chinese	110 (91.7%)	489 (76.2%)
	Minority ethnic groups	10 (8.3%)	153 (23.8%)
Nature of kindergarten	Publicly run	60 (50%)	388 (60.4%)
	Privately run	60 (50%)	254 (39.6%)
Teaching experience	Within 1 year	44 (36.7%)	102 (15.9%)
	2–5 years	54 (45%)	305 (47.5%)
	6–10 years	13 (10.8%)	173 (26.9%)
	11 years and above	9 (7.5%)	62 (9.7%)
Professional background	Early childhood education	95 (79.2%)	558 (86.9%)
	Other majors	25 (20.8%)	85 (13.1%)

Sample 2: A formal survey was conducted in January 2022 through offline random sampling, with a total of 700 paper questionnaires distributed and 662 collected. After excluding 20 invalid questionnaires, there were a total of *N* = 642 (*M*_age_ = 28.12 *SD* = 5.77, 89.7% female) valid responses, with an effective rate of 96.98%. The survey mainly focused on kindergarten teachers in ethnic minority areas such as Aba Prefecture, as well as Han areas such as Chengdu and Mianyang. Among the survey participants, 489 individuals identified as Han nationality, while 153 were from ethnic minority groups. The sample comprised 388 teachers from public kindergartens and 254 from private institutions. The teaching experience varied: 102 teachers had less than 1 year, 305 possessed 2–5 years of experience, 173 had 6–10 years under their belt, and 62 boasted over 11 years of tenure. In terms of academic focus, 558 were specialized in preschool education, and the remaining 85 teachers had majors in different fields (see Table 1). Both of the aforementioned samples were collected through the online platform.^1^ We first introduced the purpose and content of the study to the participants and assured them that the collected data would be handled confidentially.

**Figure 1:**
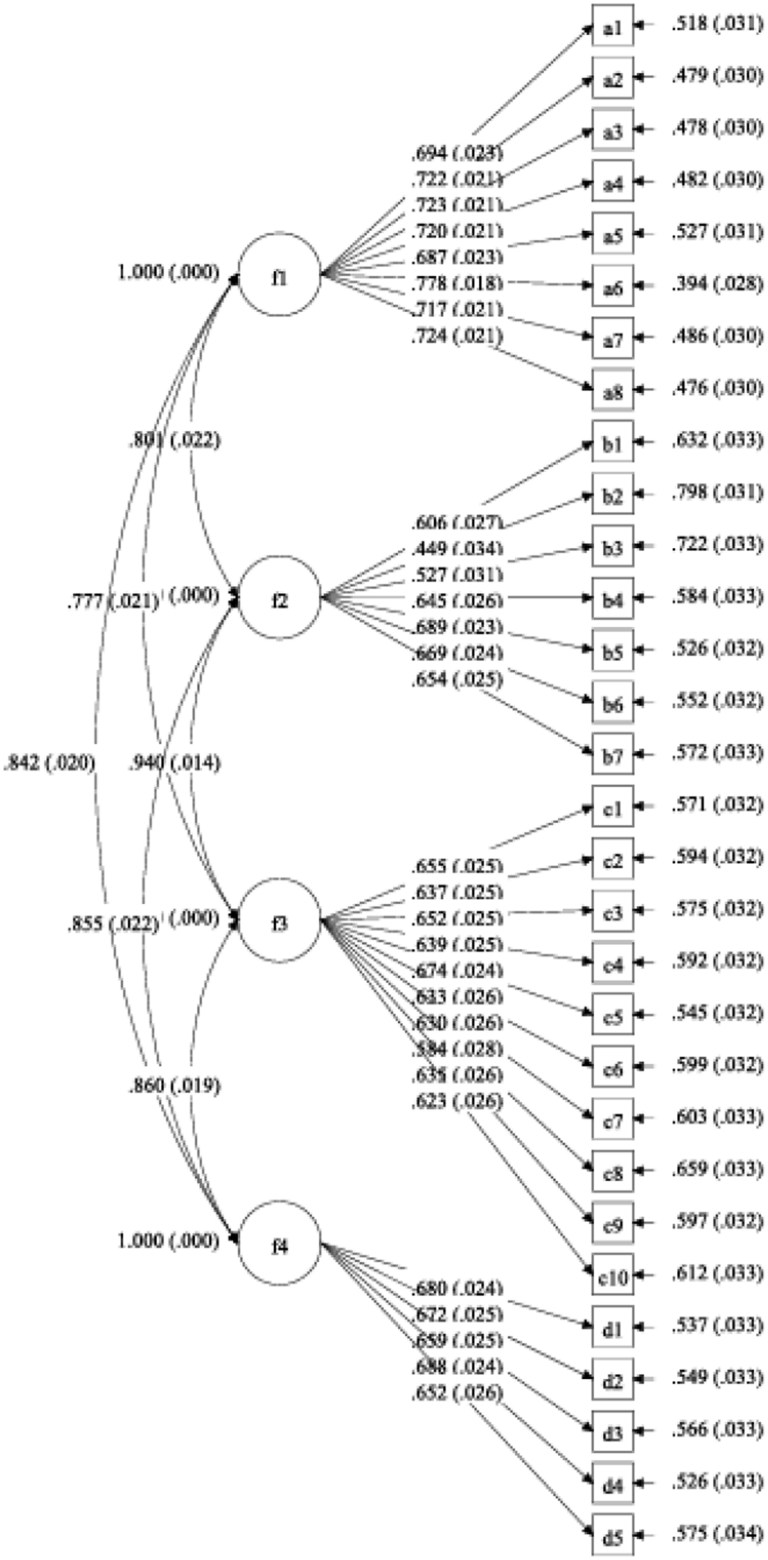
Four-factor factorial model. f1, Health concept; f2, Health abilities; f3, Healthy behaviors; f4, Healthy behaviors.

### 2.2 Preparation of questionnaire

The survey was conducted using a mixed research method that combined qualitative and quantitative approaches in three sequential stages: firstly, by reviewing relevant literature to establish an initial framework; secondly, by designing the project, conducting interviews with kindergarten teachers, and obtaining expert assessments to form a preliminary questionnaire; and finally, by evaluating the psychometric properties to develop the formal questionnaire. Both qualitative and quantitative research participants were informed of the purpose of the study, volunteered to participate, and had the right to withdraw from the study at any time.

#### 2.2.1 Determine the dimensions and items of the questionnaire

First, by referring to relevant theories of health literacy (1, 5–7), Professional Standards for Kindergarten Teachers (Trial) (23), Health Literacy of Chinese Citizens—Basic Knowledge and Skills (24), and summarizing and synthesizing literature materials, such as the open questionnaire on health literacy (11–16, 25). The initial evaluation framework for assessing the health literacy of kindergarten teachers was developed based on four dimensions: health concept, health behavior, health ability, and health knowledge.

Secondly, the dimensions of the questionnaire were determined and the questionnaire items were compiled. In order to verify the suitability of the evaluation indicators for kindergarten teachers’ health literacy, 10 kindergarten teachers were individually interviewed in this study. The existing research results and interview findings were integrated to determine the questionnaire dimensions, which included health concept, health behavior, health ability, and health knowledge. Based on the definition of health literacy and the existing measuring tools of health literacy (11–16, 25). The questionnaire items were assembled.

#### 2.2.2 Developing a predictive questionnaire

Based on the measurement tools of health literacy and the interview results, a prediction questionnaire containing 60 items was developed. Firstly, the original items were analyzed, inappropriate and ambiguous expressions were modified, and items with similar content were combined to form an initial questionnaire for kindergarten teachers’ health literacy consisting of 58 items. Secondly, two experts in preschool education and five graduate students in preschool education were asked to evaluate whether the items were reasonable. Finally, a questionnaire composed of 55 items for kindergarten teachers’ health literacy was formed. The questionnaire adopted a 5-point scoring method, requiring kindergarten teachers to evaluate from “completely inconsistent (1 point)” to “completely consistent (5 points)” according to the actual situation. The higher the total score, the higher the health literacy of kindergarten teachers.

#### 2.2.3 Form a formal questionnaire

The prediction questionnaire, consisting of 55 items, will be distributed to the prediction sample. A preliminary item analysis of the prediction results will be conducted to determine the questionnaire items and structure. Subsequently, a formal questionnaire comprising 53 items will be developed.

### 2.3 Statistical analysis

Sample 1 (*N* = 120) was utilized for item analysis and exploratory factor analysis (EFA). EFA is designed to determine the expected factors of a questionnaire. The Kaiser-Meyer-Olkin (KMO) measure assesses whether factor analysis adequately explains the shared variance among the items. If the KMO is greater than or equal to 0.8 and Bartlett’s sphericity test has a *p*-value less than 0.05, it indicates that factor analysis is appropriate (26). Sample 2 (*N* = 642) was used for confirmatory factor analysis (CFA), internal consistency reliability assessment, and split-half reliability testing of questionnaire scores. Specifically, if χ^2^/*df* ≤ 3, CFI > 0.9, TLI > 0.9, RMSEA <0.08, and SRMR <0.08, the model was acceptable (27). In addition to Cronbach’s *α*, this study also considered McDonald’s *ω* as an indicator of internal consistency. Typically, a coefficient greater than 0.7 indicates acceptability. SPSS 25.0 software was utilized to perform descriptive statistics, correlation analysis, exploratory factor analysis, internal consistency reliability, and split-half reliability analysis. Additionally, Mplus 7.0 software was employed to conduct confirmatory factor analysis.

## 3 Results

### 3.1 Project analysis

This study primarily utilizes the critical ratio method and the total correlation method to analyze the items of the questionnaire (25). First, the critical ratio method was utilized. The total health literacy score of all subjects was calculated and sorted according to the order of the questionnaire’s total score. The top 27% constituted the high group, while the bottom 27% formed the low group. Subsequently, an independent sample T-test was conducted. The results indicated that there were no statistically significant differences between the two groups in question 41 (*p* = 0.061, *p* > 0.05). However, significant differences were observed in the remaining 54 questions, with *p*-values all less than 0.05. Consequently, question 41 was excluded while retaining the remaining 54 questions. Secondly, the total correlation method was employed to identify correlations between item scores and the overall health literacy questionnaire score. If a correlation coefficient between an item and overall score was found to be insignificant and less than 0.3, then that particular question would be removed from consideration (28). The results indicated that the correlation coefficient between item 1 and the total score was less than 0.3. However, the correlation coefficient between the remaining items and the total score ranged from 0.422 to 0.757 (*p* < 0.001), suggesting that the remaining 53 items could effectively assess the health literacy level of the subjects to varying degrees.

### 3.2 Validity analysis

#### 3.2.1 Correlation between different factors and the overall questionnaire score

Based on the results presented in Table 2, it is evident that the correlation coefficient among the factors ranges from 0.463 to 0.490. This indicates that the measured directions of the factors are consistent, relatively independent, and cannot be substituted for each other. Additionally, the correlation coefficient between the total score of the kindergarten teachers’ health literacy questionnaire and the scores of the four factors ranged from 0.711 to 0.820, suggesting a high level of consistency between the contents measured by each factor and those outlined in the questionnaire (28).

**Table 2:** Correlation matrix of factors and total scores of kindergarten teachers’ health literacy questionnaire (*N* = 120).

Dimensions	Health concept	Healthy behaviors	Health abilities	Health knowledge	Total questionnaire
Health concept	1				
Healthy behaviors	0.471 ^***^	1			
Health abilities	0.490 ^***^	0.472 ^***^	1		
Health knowledge	0.469 ^***^	0.463 ^***^	0.466 ^***^	1	
Total Questionnaire	0.791 ^***^	0.820 ^***^	0.770 ^***^	0.711 ^***^	1

^***^p < 0.001.

#### 3.2.2 Structural validity

##### 3.2.2.1 Exploratory factor analysis

Sample 1 (*N* = 120) was used to conduct an exploratory factor analysis on the 53 items retained after item analysis. The results showed that the Kaiser-Meyer-Olkin measure of sampling adequacy (KMO) was 0.871, and Bartlett’s test of sphericity was significant (*p* < 0.001), indicating that the data were suitable for factor analysis. The principal component analysis method was employed to determine the number of factors based on eigenvalues greater than 1. Additionally, orthogonal rotation using the maximum variance method was conducted to eliminate items with factor loadings less than 0.5, factors with cross-loadings, and factors with fewer than three items. Following rotation, 30 items were retained and four factors were extracted, explaining 61.220% of the total variation. The factor loadings ranged from 0.571 to 0.844 (see Table 3 for specific results). In addition, Descriptive statistics for the four factors are presented in Table 4.

**Table 3:** Results of exploratory factor analysis of kindergarten teachers’ health literacy questionnaire (*N* = 120).

Item	Health concept	Healthy behaviors	Health abilities	Health knowledge
7	0.844			
5	0.812			
9	0.758			
2	0.752			
4	0.729			
3	0.719			
8	0.719			
6	0.671			
35		0.780		
36		0.777		
34		0.770		
37		0.750		
31		0.632		
33		0.602		
32		0.601		
29		0.592		
27		0.578		
26		0.571		
16			0.675	
15			0.658	
14			0.652	
20			0.650	
11			0.648	
19			0.645	
17			0.612	
51				0.736
54				0.720
44				0.720
55				0.693
53				0.575
Interpretation rate %	18.029	17.822	14.767	10.602
Cumulative interpretation rate %	18.029	35.851	50.618	61.220

**Table 4:** Descriptive statistics for the four factors in sample 1 (*N* = 120) and sample 2 (*N* = 642).

	*M*	*SD*	Medians	Skewness	Kurtosis	Mix values	Max values
Health concept	4.60	0.49	4.88	−1.25	0.78	2.88	5
	**4.21**	**0.68**	**4.25**	**−1.77**	**4.46**	**1.13**	**5**
Healthy behaviors	4.08	0.61	4.14	−0.51	0.02	2.14	5
	**3.82**	**0.62**	**3.95**	**−1.27**	**2.54**	**1**	**5**
Health abilities	4.21	0.53	4.1.	−0.23	−0.41	2.9	5
	**3.90**	**0.62**	**3.95**	**−1.46**	**3.36**	**1.2**	**5**
Health knowledge	4.31	0.63	4.40	−1.23	1.86	2.2	5
	**4.09**	**0.70**	**4.2**	**−1.48**	**3.32**	**1**	**5**

The bolded information pertains to the descriptive statistics of Sample 2.

##### 3.2.2.2 Confirmatory factor analysis

Sample 2 was used to conduct confirmatory factor analysis on the 30 retained items, which were categorized into four dimensions. The results are presented in Table 5 (Figure 1). As shown in Table 5, χ^2^/*df* = 1.945 < 3, CFI and TLI values are all above 0.9, SRMR = 0.034, and RMSEA = 0.038 < 0.08. Consequently, all the aforementioned indices satisfy the criteria, demonstrating an excellent model fit for the scale. Therefore, it is justified to affirm the validity of the model for the purposes of this research. Descriptive statistics for the four factors are presented in Table 4.

**Table 5:** Overall fitting coefficient of the confirmatory factor analysis model for the kindergarten teachers’ health literacy questionnaire (*N* = 642).

χ^2^	*df*	χ^2^/*df*	CFI	TLI	SRMR	RMSEA
764.079	399	1.945	0.956	0.952	0.034	0.038

### 3.3 Reliability analysis

“Reliability refers to the consistency of test results. A higher reliability coefficient indicates more stable and dependable test results. A questionnaire is considered to have good reliability if its reliability coefficient is above 0.7, while a coefficient below 0.35 indicates poor reliability. If the reliability does not meet the measurement standards, it is deemed unacceptable” (29). In this research, we will assess the reliability of the measurement instruments by evaluating Cronbach’s *α*, McDonald’s *ω*, and the consistency of split-half reliability across Sample 1 and Sample 2. Sample 1: the overall questionnaire has a Cronbach’s α of 0.939, with the range of Cronbach’s α for the four dimensions being from 0.822 to 0.917. In this sample, the McDonald’s *ω* coefficients also exhibit excellent performance (overall questionnaire = 0.939; range for the four dimensions = 0.827–0.918). Sample 2: the Cronbach’s α coefficient for the total questionnaire is 0.944, with each dimension ranging from 0.859 to 0.926. The McDonald’s ω coefficients for the various dimensions range from 0.803 to 0.895, and the overall questionnaire’s McDonald’s ω coefficient is 0.948. The split-half reliability coefficients for the four dimensions range from 0.864 to 0.923 (see Table 6). The above results collectively demonstrate that the questionnaire possesses good reliability.

**Table 6:** Reliability of the health literacy questionnaire for kindergarten teachers.

	Health concept	Healthy behaviors	Health abilities	Health knowledge	Total questionnaire
Cronbach’s α (*N* = 120)	0.906	0.917	0.864	0.822	0.939
McDonald’s ω (*N* = 120)	0.907	0.918	0.859	0.827	0.939
Cronbach’s α (*N* = 642)	0.917	0.926	0.904	0.859	0.944
McDonald’s ω (*N* = 642)	0.895	0.872	0.805	0.803	0.948
Split-half reliability (*N* = 642)	0.923	0.923	0.911	0.864	0.814

## 4 Discussion

Children aged 3–6 experience rapid physical growth, yet remain vulnerable and in need of attentive care. Their adaptability is limited, making them susceptible to societal influences that can impact their health. As a key setting for early learning, the kindergarten is a potential site for safety and health incidents. Given their pivotal role, teachers must possess strong health literacy to foster the comprehensive well-being of children. However, China currently lacks effective tools to measure the health literacy of kindergarten teachers. Therefore, this study confirms that the Health Literacy Questionnaire for Kindergarten Teachers, which consists of 30 items across four subscales, is a psychometrically robust measurement tool.

Additionally, this study possesses both theoretical and practical significance. In terms of theoretical significance, the questionnaire offers a corresponding theoretical framework for the current field, contributing to the body of knowledge by establishing a structured approach to assess health literacy among kindergarten teachers. The practical significance of this research is evident in its capacity to inform the design of educational programs and interventions aimed at enhancing the health literacy of kindergarten teachers. With a validated questionnaire, educators and policymakers can identify areas where additional support or training is needed, ultimately leading to improvements in both the quality of health education provided in kindergartens and the overall health and well-being of young children.

### 4.1 Questionnaire structure

Based on the existing studies on health literacy, this study developed a health literacy questionnaire for kindergarten teachers by referring to previous researchers’ work and conducting interviews with kindergarten teachers. The results show that they are consistent with the expected hypothesis. The questionnaire underwent a theoretical and statistical analysis, which led to the identification of four key dimensions: *health concepts* (8 items), *health behaviors* (10 items), *health ability* (7 items), and *health knowledge* (5 items). These dimensions were determined after comprehensive consideration of their contents and internal connections. To be more specific, the health concept dimension relates to the comprehension of kindergarten teachers regarding the essence, significance, and characteristics of health education, along with their perceived duty and responsibility in its educational implementation. The health behavior encompasses the application of accurate principles, empirical knowledge, and competencies by teachers when engaging in relevant activities. The dimension of health ability includes the foundational and specialized competencies essential for kindergarten teachers in their professional roles. The health knowledge dimension is all-encompassing, covering crucial information related to daily life and educational practices within kindergartens, which is essential for the health and development of both teachers and children. It also integrates professional understanding of physical and mental child development, an area in which kindergarten teachers should be well-versed.

This study differs from previous ones. Traditional qualitative research often posits that the health literacy of kindergarten teachers is composed of four elements: knowledge about health, information on health management, health promotion, and practical behaviors. These studies typically induce and summarize kindergarten teachers’ understanding of health literacy, which to some extent limits a deeper and broader comprehension of health literacy (10). This research overcomes this limitation by developing a questionnaire that comprehensively covers cognition, behavior, ability, and knowledge, based not only on the professional development standards of kindergarten teachers but also incorporating the teachers’ own perspectives. This approach undoubtedly offers new insights for researchers and fosters the development of kindergartens. More importantly, it provides strong support and reference for guiding the daily work of kindergarten teachers, contributing to the enhancement of their professional quality and the quality of educational practices. However, kindergarten teachers, in their daily work, although they can refer to the four-dimensional questionnaire developed in this study, which covers cognition, behavior, ability, and knowledge, should also integrate ongoing reflective criticism and professional growth. This means that teachers should continuously review and assess their teaching practices, accept feedback with an open mind, and identify and address potential issues in the educational process. Such a reflective and critical attitude is essential for teachers to remain sensitive and adaptable in the ever-changing educational environment.

### 4.2 Validity

Based on existing research, this study conducted interviews with kindergarten teachers and consulted scholars in professional fields to develop the initial questionnaire. After group testing, analysis, deletion, and modification by kindergarten teachers, the final version of the kindergarten teachers’ health literacy questionnaire was established. Therefore, the dimensions and items of the questionnaire compiled in this study accurately reflect the characteristics of kindergarten teachers’ health literacy and demonstrate good content validity. The correlation coefficients for all dimensions ranged from 0.463 to 0.490, indicating a moderate correlation; while the correlation coefficients between the four dimensions of the questionnaire and the total score ranged from 0.711 to 0.820, showing a moderately high correlation. The results of confirmatory factor analysis demonstrated that each index of the questionnaire met measurement standards, confirming its good structural validity.

The results of both exploratory factor analysis and confirmatory factor analysis indicated that the structure of the kindergarten teachers’ health literacy questionnaire was found to be stable. Specifically, the exploratory factor analysis revealed that the health concept dimension had the largest variance explanation rate for the questionnaire, accounting for 18.029%. Additionally, it was observed that the variance explanation rate of health behavior was even larger, suggesting that both health concept and health behavior play crucial roles in shaping kindergarten teachers’ health literacy. Furthermore, it is important to note that health knowledge and health ability are also significant components of kindergarten teachers’ overall health literacy.

### 4.3 Reliability

The reliability analysis of the formal questionnaire and its dimensions indicates that both Cronbach’s *α* and McDonald’s *ω* exceed 0.8 in Samples 1 and 2. This suggests that the internal consistency coefficient of the questionnaire is strong. Furthermore, the questionnaire’s split-half reliability scores range from 0.814 to 0.923, either meeting or exceeding the standard criteria for psychological assessments.

In conclusion, the differentiation, reliability, and validity of the kindergarten teachers’ health literacy questionnaire developed in this study meet established standards for measurement and can be utilized as an effective tool for assessing kindergarten teachers’ health literacy levels in academic research settings.

## 5 Limitations and future directions

This study has several limitations. Firstly, the survey subjects were primarily kindergarten teachers from the Southwest region, and the data may not be universally representative of the entire country. Future research should replicate the validation using samples from different regions and even different cultures. Secondly, the primary subjects of the survey were kindergarten teachers, and the use of self-reported questionnaires for data collection might lead to common method bias due to social desirability effect. To ensure the accuracy of the results, future studies could employ more objective measurement methods, such as peer evaluations among teachers. Finally, in the design of each questionnaire item, although we strived to ensure that each dimension’s subordinate items were representative, the applicability of some items may indeed vary due to regional and cultural differences. Therefore, future research is needed to expand the sample size and further test the applicability of each item in different regions and cultures.

## 6 Conclusion

We ultimately obtained a health literacy questionnaire for kindergarten teachers, consisting of 4 dimensions and 30 items. These four dimensions are health concepts, health behaviors, health abilities, and health knowledge. Despite the limitations mentioned above, preliminary research results indicate that this newly developed tool has good reliability and validity, making it a suitable measurement tool for assessing the health literacy of kindergarten teachers. In sum, the development of this questionnaire is not only a contribution to the individual professional development of kindergarten teachers, but also an important step in enhancing the quality of kindergarten education and promoting the development of health education.

## Data Availability

The raw data supporting the conclusions of this article will be made available upon reasonable request to Han Ying, hanying@abtu.edu.cn.

## Footnotes

^1^ https://www.wjx.cn/
